# The Effect of Angiotensin Converting Enzyme (ACE) I/D Polymorphism on Atherosclerotic Cardiovascular Disease and Cardiovascular Mortality Risk in Non-Hemodialyzed Chronic Kidney Disease: The Mediating Role of Plasma ACE Level

**DOI:** 10.3390/genes13071121

**Published:** 2022-06-23

**Authors:** Hendri Susilo, Budi Susetyo Pikir, Mochammad Thaha, Mochamad Yusuf Alsagaff, Satriyo Dwi Suryantoro, Citrawati Dyah Kencono Wungu, Ifan Ali Wafa, Cennikon Pakpahan, Delvac Oceandy

**Affiliations:** 1 Doctoral Program of Medical Science, Faculty of Medicine, Universitas Airlangga, Surabaya 60132, Indonesia; hendrisusilo@staf.unair.ac.id; 2 Department of Cardiology and Vascular Medicine, Universitas Airlangga Hospital, Surabaya 60115, Indonesia; yusuf_505@fk.unair.ac.id; 3 Department of Cardiology and Vascular Medicine, Faculty of Medicine, Universitas Airlangga, Surabaya 60132, Indonesia; 4 Department of Internal Medicine, Faculty of Medicine, Universitas Airlangga, Surabaya 60132, Indonesia; satriyo.dwi.suryantoro@fk.unair.ac.id; 5 Department of Internal Medicine, Universitas Airlangga Hospital, Surabaya 60115, Indonesia; 6 Department of Physiology and Medical Biochemistry, Faculty of Medicine, Universitas Airlangga, Surabaya 60132, Indonesia; 7 Institute of Tropical Disease, Universitas Airlangga, Surabaya 60286, Indonesia; 8 Faculty of Medicine, Universitas Airlangga, Surabaya 60132, Indonesia; ifan.ali.wafa-2016@fk.unair.ac.id; 9 Department of Biomedicine, Faculty of Medicine, Universitas Airlangga, Surabaya 60132, Indonesia; cennikon.pakpahan@fk.unair.ac.id; 10 Division of Cardiovascular Science, Manchester Academic Health Science Centre, University of Manchester, Manchester M13 9PG, UK; delvac.oceandy@manchester.ac.uk

**Keywords:** chronic kidney disease, gene polymorphism, angiotensin-converting enzyme, cardiovascular disease, mortality risk

## Abstract

The association between angiotensin-converting enzyme insertion/deletion (ACE I/D) polymorphisms and plasma ACE levels may allow for the optimization of a preventive intervention to reduce cardiovascular morbidity and mortality in the chronic kidney disease (CKD) population. In this study, we aimed to analyze the association between ACE I/D polymorphism and cardiovascular mortality risk among non-hemodialyzed chronic kidney disease patients. This cross-sectional study examined 70 patients of Javanese ethnic origin with stable CKD who did not receive hemodialysis. ACE I/D polymorphisms, plasma ACE levels, atherosclerotic cardiovascular disease (ASCVD) risk, and cardiovascular mortality risk were investigated. As per our findings, the I allele was found to be more frequent (78.6) than the D allele (21.4), and the DD genotype was less frequent than the II genotype (4.3 vs. 61.4). The ACE I/D polymorphism had a significant direct positive effect on plasma ACE levels (path coefficient = 0.302, *p* = 0.021). Similarly, plasma ACE levels had a direct and significant positive effect on the risk of atherosclerotic cardiovascular disease (path coefficient = 0.410, *p* = 0.000). Moreover, atherosclerotic cardiovascular disease risk had a significant positive effect on cardiovascular mortality risk (path coefficient = 0.918, *p* = 0.000). The ACE I/D polymorphism had no direct effect on ASCVD and cardiovascular mortality risk. However, our findings show that the indirect effects of high plasma ACE levels may be a factor in the increased risk of ASCVD and cardiovascular mortality in Javanese CKD patients.

## 1. Introduction

Chronic kidney disease is defined by the presence of kidney damage or decreased kidney function for at least three months, with pathologic anomalies in the kidney or reduced glomerular filtration rate (GFR) [1]. CKD is a global health burden that affects 8–16% of the worldwide population [2]. This condition could result in significant end-stage renal disease (ESRD) as well as an increase in the risk of cardiovascular disease [3]. Rather than abnormal kidney functions, cardiovascular complications have been determined as the main causes of death in this high-risk population. CKD generates a systemic, chronic pro-inflammatory condition that contributes to vascular and myocardial remodeling [4]. The increased mortality risk in CKD patients could be influenced by common risk factors, such as hypertension and diabetes [5]. Cardiovascular disease and CKD are closely related, as a disease in one organ can lead to dysfunction, which could ultimately result in the failure of both organs [6].

In addition, non-traditional risk factors such as oxidative stress and inflammation also contribute to the high prevalence of CKD [7,8]. Chronic kidney disease is linked to homeostatic deregulation of soluble molecule synthesis, release, and degradation, as well as immune system disturbances caused by disruption of cytokines and inflammatory mediators, and decreased renal clearance, which results in higher levels of circulating cytokines [9]. Oxidative stress plays a significant role in the progression and death of CKD patients. The increase of inflammatory cytokines and other inflammatory markers has been demonstrated to be an independent predictor of cardiovascular outcomes in patients with CKD [10,11]. The inflammatory process is closely related to the renin-angiotensin-aldosterone system (RAAS). The increased activity of angiotensin II through the angiotensin II type I receptor (AT1R) can cause inflammation and the release of interleukin-6 (IL-6) and decrease in nitric oxide (NO) activity, causing endothelial dysfunction and increasing the risk of atherosclerosis [12]. Increased levels of angiotensin II could not be separated from the role of the angiotensin-converting enzyme (ACE), which functions to convert angiotensin I to angiotensin II [13].

Several studies have reported associations between ACE gene polymorphisms (insertion/deletion) and cardiovascular diseases, including endothelial dysfunction, atherosclerosis, and heart failure [14,15,16]. The ACE I/D polymorphism has been linked to plasma ACE activity [17,18]. Previous studies showed that ACE levels are almost twofold higher in individuals with the DD genotype than in those with the II genotype, while subjects with the ID genotype have medium ACE levels [18,19,20]. The increased plasma ACE level may result in an increase in the expression levels of interleukin-6 (IL-6) and kallikrein (KLK1), which subsequently increases coronary plaque vulnerability, ulceration, and thrombosis, leading to an increased risk and mortality of cardiovascular diseases [17]. Notwithstanding, prior research on the role of the ACE I/D polymorphism in the development of cardiovascular complications in CKD patients yielded contradictory findings [21].

Up to this point, there has been a dearth of detailed information on the effect of ACE I/D polymorphisms on non-hemodialyzed CKD patients. The high prevalence of CKD with cardiovascular complications allows researchers to perform an earlier assessment to evaluate the risk of atherosclerotic cardiovascular disease (ASCVD) and cardiovascular mortality. As the existing AHA/ACC guidelines and ESC risk prediction models (Pooled Cohort Equation (PCE) and Systematic Coronary Risk Evaluation (SCORE)) were deemed insufficient for estimating cardiovascular risk and mortality in chronic kidney disease [22,23], Matsushita et al. added parameters for estimated GFR (eGFR) and albuminuria to their “CKD patch” score, which was thought to allow for more accurate calibration of cardiovascular risk in chronic kidney disease [24].

The intertwining roles between ACE I/D polymorphisms, plasma ACE level, cardiovascular disease, and mortality risk in CKD need to be investigated further to determine that preventive intervention with optimal medical therapy can be given earlier; thus, cardiovascular morbidity and mortality in the CKD population can be reduced. There has been no study to prove such associations, especially in non-hemodialyzed patients. Therefore, in this research, we aimed to determine the relationship between ACE I/D polymorphism and cardiovascular mortality risk in non-hemodialyzed CKD.

## 2. Materials and Methods

### 2.1. Study Design

This study has examined the effect of ACE gene polymorphisms and ACE levels on atherosclerotic cardiovascular disease and cardiovascular mortality in non-hemodialyzed CKD patients. This was an analytical observational study with a cross-sectional design conducted from May 2021 to December 2021. In total, 70 patients were included in this study from the outpatient clinic at Universitas Airlangga Hospital, Surabaya, Indonesia. The local ethics committee has ethically approved this study (146/KEP/2021). All individuals who accepted to participate in this study were given a comprehensive explanation of the study before it commenced, and written informed consent was obtained from all subjects who agreed to participate.

### 2.2. Sample Criteria and Data Collection

The inclusion criteria were as follows: CKD patients aged 40–79 years (according to the provisions in the ASCVD risk application), of Javanese ethnicity, stable CKD, and those who had not undergone hemodialysis. Meanwhile, this study excluded patients with acute coronary syndrome (STEMI, NSTEMI, and unstable angina), a history of acute coronary syndrome or stroke, acute heart failure, severe infection (sepsis), uncontrolled arrhythmias, metabolic syndrome acidosis, hyperkalemia, and those under treatment with ACE inhibitors and/or statins. The OMRON Professional Blood Pressure Monitor HBP-1120 was used to measure blood pressure (Omron Corporation, Osaka, Japan). History-taking and physical examination included gender, age, ethnicity, body mass index (BMI), blood pressure, and medical history (of risk factors and diseases before the study, such as diabetes, smoking, hypertension, ACS, stroke, uncontrolled arrhythmia, and heart failure). A baseline laboratory examination was performed on blood and urine samples to measure the albumin-creatinine ratio (ACR), high-density lipoprotein (HDL), and total cholesterol.

**Figure 1:**
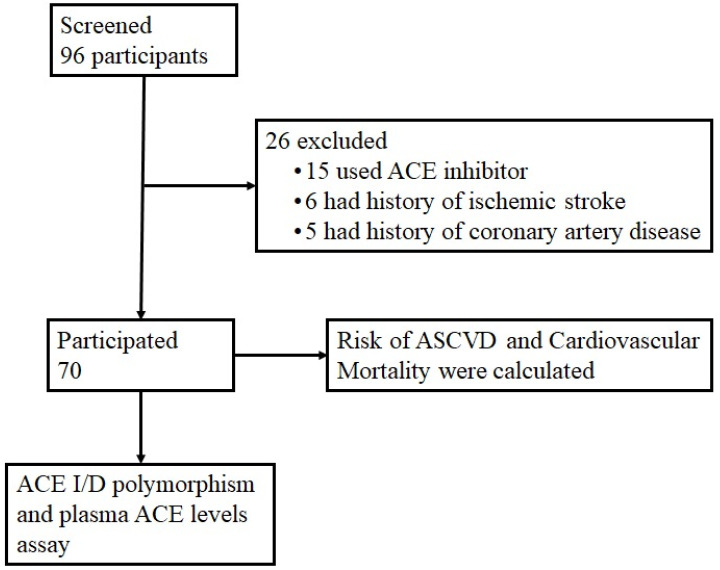
Flow chart for this cross-sectional study. There were 96 participants during the study period between May 2021 and December 2021. Twenty-six participants were not included for reasons including: under treatment with ACE inhibitor (15), had history of ischemic stroke (6), and had history of coronary artery disease (5). A total of 70 participants were included in this study. There were no statistically significant differences when comparing men and women included in this study. The risk of ASCVD and cardiovascular mortality were calculated with CKD patch. Blood plasma from the participants were used to identify ACE I/D polymorphism and measure plasma ACE level.

The ASCVD risk score (CKD patch) as a percentage estimate for a 10-year atherosclerotic cardiovascular disease risk was calculated using the following factors: eGFR, ACR, age, gender, race, blood pressure, antihypertensive use, HDL, total cholesterol, history of diabetes, and history of smoking. The CKD patch score algorithm (https://ckdpcrisk.org/ckdpatchscore/ accessed on 10 November 2021) was also used to calculate the 10-year risk of cardiovascular death, including eGFR, ACR, age, gender, systolic blood pressure, total cholesterol, and smoking status. The Chronic Kidney Disease Prognosis Consortium 2 was used to develop the CKD patch equation (https://ckdpcrisk.org/ckdpatchpce/ accessed on 10 November 2021) [24]. The flow chart of the sample collection is presented in Figure 1.

### 2.3. DNA Isolation

Approximately 5 mL of venous blood was drawn from each subject, and this was put into a venoject tube with an EDTA anticoagulant. All procedures for ACE I/D genotyping and plasma ACE measurement were performed at the Institute of Tropical Disease (ITD), Universitas Airlangga, Surabaya, Indonesia. Peripheral blood mononuclear cells (PBMC) were extracted from each sample, and DNA was extracted using the QIAamp DNA extraction kit (Qiagen, Inc., Hilden, Germany) with Cat. No. 51104, using the working procedure according to the kit. 

### 2.4. Genotyping of ACE I/D Polymorphism

Polymerase chain reaction sequence-specific primer (PCR-SSP) test was conducted to determine ACE I/D genotype using Promega GoTaq Green (Cat. No. M7122) as the master mix. The amplification reaction used a forward primer 5′CTGGAGACCACTCCCATCCTTTCT 3′ and a reverse primer 5′GATGTGGCCATCACATTCGTCAGAT 3′. The thermal cycling conditions were modified from Singh et al. [25]. The process was started with an initial denaturation at 94 °C for 3 min, followed by 40 cycles of denaturation (94 °C–30”), annealing (59.8 °C–30”), and extension (72 °C–45”). The amplification process ended with a final extension of 72 °C for 10 min. The PCR product on 2% agarose gel showed 490 bp for the II genotype (300 bp insertion), 190 bp for the DD genotype, and two bands (490 and 190 bp) for the ID genotype when visualized under the ultraviolet. Some PCR-SSP products were confirmed by direct sequencing. We performed direct sequencing for ACE gene by cutting the electrophoresis gel of the PCR-SSP products. DNA purification was carried out using the QIA quick gel extraction kit DNA purification kit with Cat. No. 28704 from Qiagen, Germany according to the manufacturer’s procedure. DNA labeling was then carried out by making a mixture of reverse primer mentioned above, Ready Reaction Premix 2.5X, BigDye Terminator Sequencing Buffer, distilled water, and DNA purification product. The Thermal Cycler was set to a temperature of 96 °C for 3 min for 25 cycles. Each cycle consisted of denaturation for 10 s at a temperature of 96 °C, annealing for 5 s at a temperature of 50 °C, and elongation for 4 min at a temperature of 60 °C. After the cycle was complete, the mixture was cooled down to 4 °C. SAM™ Solution (Lot. No.1410050) and XTerminator™ Solution (Cat. No.4376486) were mixed with the labeling product in an eppendorf tube. DNA sequencing was carried out using the ABI Prism 3500× L Genetic Analyzer 24 capillaries after the sample had been prepared.

### 2.5. Plasma ACE Levels

The plasma ACE levels were analyzed via enzyme-linked immunoassay (ELISA) procedure using Human ACE (angiotensin Ⅰ-converting enzyme) ELISA Kit (Cat. No.: E-EL-H6001, Elabscience, Houston, TX, USA) according to the manufacturer’s instructions. Samples and standards were transferred to 96-well microplates pre-coated with specific antibodies and incubated for 1 h. After the plates were washed and decanted, biotinylated detection antibodies were added to each well and incubated for 60 min. Then, an avidin-horseradish peroxidase (HRP) conjugate was added to each well, and the plates were thereafter incubated for 30 min. A substrate reagent was added, and the plates were incubated for 15 min. The stop solution was then added to each well, and the absorbance was read at 450 nm using the ELISA Humareader.

### 2.6. Data Analysis

Statistical analysis was performed using the SPSS statistics software, version 23 (IBM Corp, Armonk, NY, USA). The mean standard deviation (SD) and percentage frequency were used to analyze the data. A normality test was performed on the numerical data using the Shapiro–Wilk test. Depending on the variables, Kruskal–Wallis with Dunn’s post-hoc test or one-way ANOVA with LSD post hoc-tests were performed to assess the significance of each CKD stage. Correlational analyses between variables were analyzed using the Spearman rank-order correlation test. The observed genotypes and allele frequencies were compared with those expected to verify the Hardy–Weinberg equilibrium. The path analysis between ACE polymorphisms, plasma ACE level, ASCVD risk, and cardiovascular mortality risk was calculated using SmartPLS 3.3.7 (GmbH Company, Oststeinbek, Germany). A *p*-value less than 0.05 was used to denote statistical significance.

## 3. Results

This study has examined 70 individuals from the Airlangga University Hospital in Surabaya, Indonesia, who were 40–70 years old and have stage II–V non-hemodialyzed CKD. Table 1 provides an overview of the demographic and clinical characteristics of the research participants. In this study, the proportion of male CKD patients (52.9%) was similar to that of female CKD patients (47.1%). Except for serum creatinine, eGFR, and urine ACR, most variables in this study did not differ between each CKD stage (*p* > 0.05). This study identified multiple risk factors for atherosclerotic cardiovascular disease. The majority of the CKD patients in this study (57.1%) had diabetes and hypertension (75.7%). Moreover, most of them do not smoke tobacco products (70%). The CKD patients in this study were determined to have stage 1 to 5, with stage 3 having the largest distribution (51.43%).

For a 10-year period, differences were noted in terms of atherosclerotic cardiovascular disease and mortality risks, as shown in Table 2. In this study, non-hemodialyzed CKD patients had a significant risk of atherosclerotic cardiovascular disease (23.54%) and a very high risk of cardiovascular mortality (16.3%).

**Figure 2:**
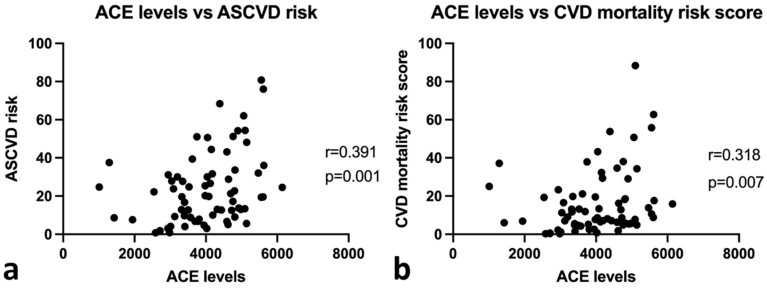
Correlations between ACE levels with ASCVD risk score and cardiovascular mortality score with Spearman analysis. (**a**) Correlation between ACE levels with ASCVD risk score. (**b**) Correlation between ACE levels with cardiovascular mortality score.

As shown in Table 3, statistical analysis reveals significant positive correlations between age, systolic blood pressure, smoking history, serum creatinine, ACR, and plasma ACE level with both atherosclerotic cardiovascular disease risk and cardiovascular mortality risk (*p* < 0.05). Significant negative correlations were also observed between HDL levels and the risk of atherosclerotic cardiovascular disease (r = −0.337, *p* = 0.004) and eGFR levels with the risk of cardiovascular mortality (r = −0.284, *p* = 0.017). A significant positive correlation was also found between CKD stage and cardiovascular mortality risk (r = 0.308, *p* = 0.009), as well as between atherosclerotic cardiovascular disease risk and cardiovascular mortality risk (r = 0.922, *p* = 0.000). Additionally, as shown in Figure 2a,b, plasma ACE levels showed a significant positive correlation with ASCVD risk (r = 0.391, *p* = 0.001) and CVD mortality risk score (r = 0.318, *p* = 0.007).

As shown in Table 4, additional data analysis of the correlation between plasma ACE levels and ASCVD and cardiovascular mortality risk in each CKD group revealed that in the severe CKD group, there was a significant correlation between plasma ACE levels and ASCVD and cardiovascular mortality risk. Meanwhile, only a significant correlation was found between plasma ACE levels and ASCVD risk in the mild to moderate CKD group. This suggests that plasma ACE plays a larger role in the risk of ASCVD and cardiovascular mortality, particularly in patients with a lower glomerular filtration rate (eGFR < 30 mL/min/1.73 m^2^).

**Figure 3:**
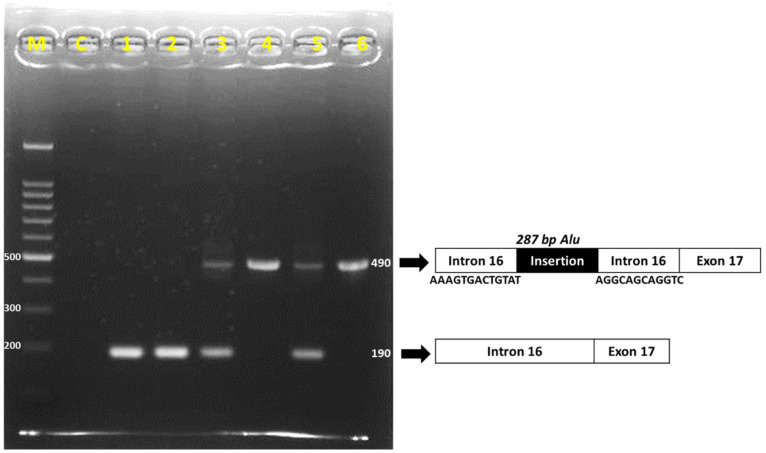
Electrophoresis of PCR-SSP products on 2% agarose gel. M = marker; C = negative control; 1 and 2 = DD genotype; 3 and 5 = ID genotype; 4 and 6 = II genotype.

**Figure 4:**
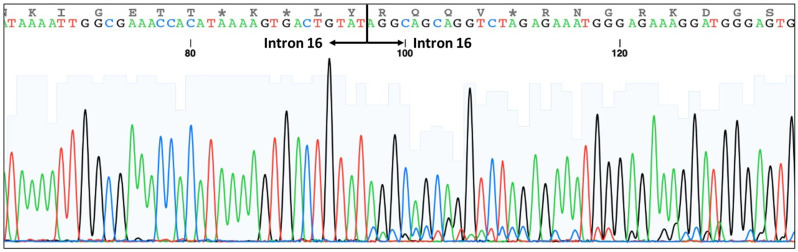
Sequencing results show that samples with DD genotype had deletions in intron 16.

The ACE I/D polymorphism (rs4646994) was successfully detected in all participants via PCR-SSP method. The II genotype showed a 490 bp band on PCR-SSP, while the DD genotype showed a 190 bp band; the ID genotype showed both 490 and 190 bp fragments (Figure 3). The sequencing result also confirmed that samples with DD genotypes had 287 bp of intron 16 deletion (Figure 4).

In Table 5, the genotype and allele frequencies of the two polymorphisms can be observed. Most samples had major homozygous genotype II (64.1%), the rest had heterozygous genotype ID (34.3%), and only a few had minor homozygous genotype DD (4.3%). It was also found that the allele frequency distribution of ACE I/D polymorphism was consistent with Hardy–Weinberg law (x^2^ = 0.023, *p*-value = 0.989).

**Figure 5:**
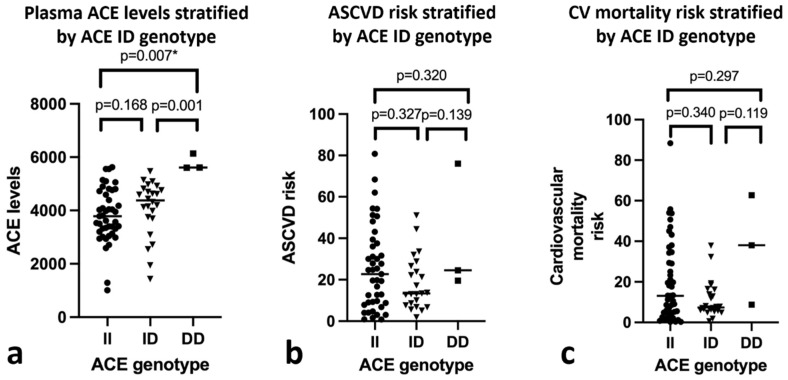
Analysis of ACE levels, ASCVD risk, and cardiovascular mortality risk between different ACE I/D genotypes (Kruskal–Wallis with Mann–Whitney post-hoc test). (**a**). Patients with DD genotype displayed higher plasma ACE levels compared to II or ID genotypes. (**b**). No difference was observed in ASCVD risk between the three genotypes. (**c**). No difference was observed in cardiovascular mortality risk between the three genotypes. * = significant at *p* < 0.05.

**Figure 6:**
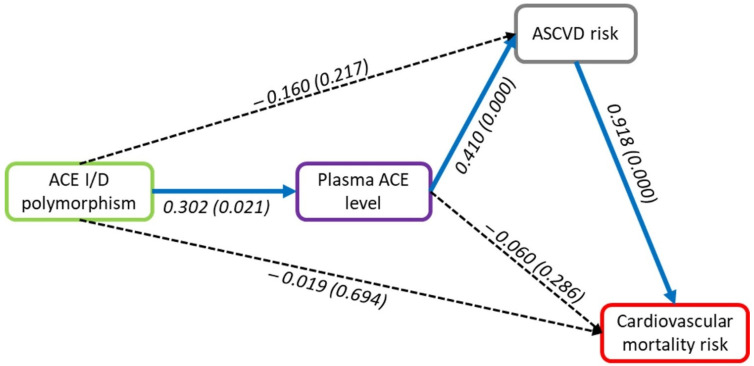
Relationship between ACE I/D polymorphism, plasma ACE level, atherosclerotic cardiovascular diseases risk, and cardiovascular mortality risk showed with path coefficient (*p*-value) on each pathway.

As shown in Figure 5, increase in ACE levels was observed in patients with the DD genotype, compared to the II or ID genotype, while there were no differences in ASCVD risk or cardiovascular mortality risk between the II, ID, and DD genotypes. To assess the relationship between ACE I/D polymorphism with plasma ACE level, atherosclerotic cardiovascular disease risk, and cardiovascular mortality risk, as illustrated in Figure 6, we performed structural equation modeling analysis using SmartPLS software. As indicated in Table 6, ACE I/D polymorphism (path coefficient = 0.302, *p* = 0.021) was determined to have a significant direct positive effect on plasma ACE level. There was also a significant direct positive effect on atherosclerotic cardiovascular disease risk from plasma ACE level (path coefficient = 0.410, *p* = 0.000). Moreover, atherosclerotic cardiovascular disease had a significant positive effect (path coefficient = 0.918, *p* = 0.000) on cardiovascular mortality risk. The ACE I/D polymorphism had no significant direct association with atherosclerotic cardiovascular disease risk but had a significant positive indirect effect on atherosclerotic cardiovascular disease risk via ACE plasma level. The indirect effect of the ACE I/D polymorphism (path coefficient = 0.124, *p* < 0.05) indicates that the ID/DD genotype had a higher ACE plasma level and atherosclerotic cardiovascular disease risk, which led to increased cardiovascular mortality risk.

## 4. Discussion

While several risk factors for atherosclerotic cardiovascular disease have been established beyond reasonable doubt, the complex interplay of genetic factors has intrigued and piqued considerable interest. The ACE gene polymorphism is still being investigated to determine whether it can be of use in predicting the extent of cardiovascular risk and mortality. In the RAAS, ACE is known to play a critical role in regulating the pathological condition, and inhibiting it has a significant therapeutic benefit [26,27].

In this cross-sectional study, our results demonstrated that the ACE I/D polymorphism had no significant effect on ASCVD or cardiovascular mortality risk in non-hemodialyzed CKD patients of Javanese ethnic origin. However, further analysis revealed that the ACE I/D polymorphism can directly affect plasma ACE levels, which are subsequently associated with higher ASCVD risk, which, in turn, could lead to increased cardiovascular mortality risk. These findings highlight the indirect relationship between the ACE I/D polymorphism and cardiovascular mortality risk mediated by plasma ACE levels. Intriguingly, the indirect effect of the ACE I/D polymorphism indicates that the ID and the DD genotypes may contribute to higher plasma ACE levels. This finding is consistent with prior studies, denoting that ACE I/D polymorphism is responsible for elevated ACE concentrations in plasma [17,19,28,29]. The ACE DD genotype was found to have a key role in altering plasma ACE levels or activity, as well as increasing the instability of atherosclerotic plaques, according to substantial data [30,31,32]. Moreover, ample evidence exists for the association of the ACE I/D polymorphism with various cardiovascular disorders, such as essential hypertension [33], coronary artery disease [32], myocardial infarction [34], and myocardial hypertrophy [35].

When repeatedly measured in the same individuals, the stability of circulating plasma ACE levels may allow the factors influencing its long-term regulation to be examined [36,37]. Plasma ACE levels have previously been found to vary significantly between individuals due to genetic factors [38]. More than half of the variance in plasma ACE levels is under the influence of the I/D polymorphism. ACE levels in individuals with the DD genotype were approximately twice than that in individuals with the II genotype. In contrast, individuals with the ID genotype had an intermediate ACE level, indicating codominance [39]. In preclinical studies, elevated plasma ACE activity was determined to accelerate the progression of atherosclerosis [40]. It has been established that angiotensin II (Ang-II), formed by cleavage of angiotensin I (Ang-I) by ACE, can bind to Ang-II type 1 (AT1R) or Ang-II type 2 (AT2R) receptors. Through ACE2, Ang-II is converted to angiotensin 1–7 (Ang-1–7), which classically interacts with the Mas receptor (MasR). Furthermore, through its interaction with AT1R, Ang-1–7 can bind to AT2R and activate the arrestin pathway. Ang-1–7 can also be synthesized from Ang-I via neprilysin (NEP). This suggests that the ACE/Ang-II/AT1R pathway can induce atherosclerosis, which would then account for the correlation between plasma ACE levels and the risk of ASCVD [41]. Nevertheless, previous clinical studies generated inconsistent findings [42,43,44,45]. This could result from multiple interactions between genetic and environmental factors, such as lifestyle, disease severity, and the presence of different sexes in each study population. It was also discovered that plasma ACE levels play a role in linking ACE I/D polymorphism with the incidence of ASCVD risk, which directly contributes to cardiovascular mortality risk. The absence of a direct association between the ACE I/D polymorphism and cardiovascular mortality risk suggests that there may be additional factors affecting plasma ACE levels (other than the ACE I/D polymorphism), such as interactions with other genes or environmental factors. Given the limited available data, we hypothesize that these factors will reduce the polymorphism’s direct correlation with CVD risk. This finding is consistent with a large cohort study involving 6714 participants that examined the relationship between ACE I/D polymorphism and cardiovascular morbidity and mortality, wherein no direct association was determined between polymorphisms and myocardial infarction, significant morbidity, or risk factor for death in CKD patients [46]. The ACE I/D polymorphism is noteworthy in that it is located within an intron. As a result, its effect on mortality could be attributed to another (functional) variant within or close to the ACE gene that is in linkage disequilibrium with the I/D polymorphism [47].

The distribution of ACE genotypes has been noted to vary significantly between ethnic groups worldwide [48]. Prior research has established that populations from a particular region of the world would exhibit a similar ACE I/D polymorphism pattern to populations from another region [49]. Studies conducted among western populations in France [50], Germany [51], Egypt [52], Spain [53], and Italy [54] found that the frequency of the D allele is more dominant than the I allele, whereas studies conducted among Asian populations in India [55], Thailand [56], and Korea [57] found that the I allele frequency is more dominant than the D allele. In line with these previous studies, we discovered that the Javanese ethnicity, who were the subjects of this current study, had a dominant I allele frequency (78.6) over the D allele (21.4), and the DD genotype was less frequent than the II genotype (4.3 vs. 61.4). This corroborates the observed similarity in allele frequencies between Asian and Western populations. Earlier research among the Indonesian population revealed a similar pattern [58,59]. Additionally, the genotypic distribution of the total population in this current study was consistent with the Hardy–Weinberg equilibrium, indicating that the population’s genotype and allele frequencies have remained constant over several successive generations, ruling out the presence of population stratification as a study bias. It is thus very important to study ACE I/D polymorphisms in genetically homogeneous populations.

Albeit the presence of the ACE I/D polymorphism genotype has been hypothesized to influence response to ACE inhibitor treatment in the past, results of several studies have been contradictory. Prior research in the Italian [60], Iranian [61], and Malaysian [62] populations demonstrated that ACE inhibitors have a greater health benefit in carriers of the DD genotype than ID or II genotypes. In contrast, earlier studies have shown that carriers of the II genotype have a better response to ACE inhibitor therapy than those carrying the DD genotype [63,64]. Indeed, other studies have demonstrated that the ACE I/D polymorphism genotype does not affect ACE inhibitor adherence [65,66]. As evidence for this finding remains limited due to the scarcity of comprehensive pharmacogenetic studies, it is far too early to draw solid conclusions with regard to the optimal ACE inhibitor treatment for different genotypes. 

The results of this study indicate that plasma ACE level has an important role in the risk of ASCVD and cardiovascular mortality, in which plasma ACE will affect inflammation, oxidative stress, endothelial dysfunction, and vascular and myocardial remodeling [67]. Therefore, preventive efforts through pharmacological interventions are important to reduce cardiovascular morbidity and mortality. Among the drugs that can be administered to patients with non-hemodialysis CKD are ACE inhibitors, which have cardiorenal protection properties that have been shown to reduce adverse cardiovascular and renal events, and all-cause mortality in stage 3–5 non-dialysis CKD patients [68]. In addition, statin drugs can also be given according to the patient’s cardiovascular risk stratification and CKD stage. This is in accordance with the recommendations issued by the 2021 ESC Guidelines on cardiovascular disease prevention in clinical practice [69]. On the other hand, since the administration of ACE inhibitors and statins could act as confounding factors which could reduce ASCVD and cardiovascular mortality risk, we had excluded the patients who were under treatment with ACE inhibitors and statins from this study.

To our knowledge, this is the first comprehensive study of the ACE I/D polymorphism in non-hemodialyzed CKD patients of Javanese ethnicity, Indonesia’s largest ethnic group, in relation to ASCVD and cardiovascular mortality risk. The importance of plasma ACE levels as a bridge connection between the ACE I/D polymorphism and ASCVD and cardiovascular mortality risk was also demonstrated using path analysis in this study. As a result, determining patients’ genotypes may be able to reduce the extent of cardiovascular risk and mortality trends in the CKD population, as the ACE I/D polymorphism described in this study provides a possible tool for patient prevention and early intervention treatment. Although this current study was conducted in very homogeneous communities, the sample size was relatively small, thus potentially limiting its statistical significance. However, the sample size was large enough to find associations between ACE I/D polymorphism, ACE plasma level, and ASCVD/cardiovascular mortality risks. We have also calculated the post-hoc statistical power by online post-hoc power calculator https://clincalc.com/stats/Power.aspx accessed on 10 June 2022. and found that with the present sample size our study still had strong power.

## 5. Conclusions

As per our findings, based on the allele and its genotype, it can be concluded that the ACE I/D polymorphism is not associated with ASCVD and cardiovascular mortality risk. Direct effects of the increase in plasma ACE levels further underlie the mediating mechanism of increasing ASCVD and cardiovascular mortality risk among Javanese CKD patients. The I allele predominated in the majority of the participants in this investigation. Thus, our finding supports a similar pattern among Asian populations, especially in Indonesia. While more research in different populations and ethnicities is needed to determine the clinical significance of these findings, the presence of the ACE I/D polymorphism and ACE plasma level in Javanese non-hemodialyzed CKD patients may serve as predictive markers for ASCVD and cardiovascular mortality risk. Thus, it may also apply to other Asian countries.

## Figures and Tables

**Table 1:** Basic characteristics of the participants.

Variable	CKD Stage				All Patients (*n*= 70)	*p*
	Stage 2 (*n* = 3)	Stage 3 (*n* = 36)	Stage 4 (*n* = 20)	Stage 5 (*n* = 11)		
Gender, male%	3 (100)	19 (52.8)	11 (20)	4 (36.4)	37 (52.9)	0.271 ^A^
Age (years)	58.0 ± 12.17	58.47 ± 5.92	58.65 ± 6.39	54.64 ± 10.62	57.90 ± 7.19	0.716 ^B^
Diabetes *n* (%)	3 (100)	28 (77.8)	15 (75)	8 (72.7)	40 (57.1)	0.784 ^A^
Hypertension *n* (%)	3 (100)	32 (88.9)	16 (80)	10 (90.9)	53 (75.7)	0.866 ^A^
Smoking history						0.521 ^A^
Non-smoker *n* (%)	2 (66.7)	26 (72.2)	12 (60)	9 (81.8)	49 (70.0)	
Current smoker *n* (%)	0 (0)	1 (2.8)	3 (15)	0 (0)	4 (5.7)	
Former smoker *n* (%)	1 (33.3)	9 (25)	5 (25)	2 (18.2)	17 (24.3)	
Body mass index (kg/m^2^)	28.32 ± 5.45	26.04 ± 5.42	27.09 ± 5.28	24.39 ± 4.48	26.18 ± 5.23	0.387 ^B^
Systolic blood pressure (mmHg)	134.33 ± 3.06	145.44 ± 23.33	141.95 ± 21.95	147.27 ± 28.33	144.26 ± 23.09	0.800 ^C^
Diastolic blood pressure (mmHg)	79.33 ± 4.73	81.42 ± 12.33	81.30 ± 10.51	81.27 ± 15.58	81.27 ± 11.98	0.887 ^B^
Total cholesterol (mg/dL)	192.0 ± 52.72	181.22 ± 52.44	184.25 ± 52.23	184.09 ± 47.75	183.00 ± 50.63	0.902 ^B^
High-density lipoprotein (mg/dL)	37.0 ± 1.73	43.75 ± 15.43	36.65 ± 5.79	34.18 ± 7.36	39.93 ± 12.42	0.022 ^B^
Serum creatinine (mg/dL)	1.3 ± 0.04	1.71 ± 0.27	2.92 ± 0.61	5.46 ± 2.21	2.63 ± 1.63	0.000 *^,B^
eGFR (mL/min/1.73 m^2^)	63.67 ± 3.79	40.94 ± 7.27	21.70 ± 4.13	11.09 ± 3.33	31.73 ± 14.81	0.000 *^,B^
Urine ACR (mg/g)	28.19 ± 31.28	313.7 ± 475.92	590.53 ± 777.54	1805.69 ± 1352.2	615.01 ± 913.74	0.000 *^,B^
Plasma ACE (pg/mL)	3417.83 ± 546.8	3908.79 ± 1158.18	4151.9 ± 1134.18	4145.85 ± 752.48	3994.46 ± 1074.47	0.637 ^B^

* *p* < 0.05. ^A^ = chi-square test; ^B^ = Kruskal–Wallis with Dunn’s post-hoc test; ^C^ = ANOVA with LSD post-hoc test. ACR = albumin/creatinine ratio; eGFR = estimated glomerular filtration rate; ACE = angiotensin-converting enzyme.

**Table 2:** Atherosclerotic cardiovascular disease and mortality risk scores according to CKD patch.

Variable	Minimum	Maximum	Mean	Std. Deviation
Ten-year risk of atherosclerotic cardiovascular disease (%)	0.8	80.8	23.54	18.79
Ten-year risk of cardiovascular mortality (%)	0.3	88.4	16.3	17.02

**Table 3:** Results of the correlational analysis between variables in this study.

Variables	Risk of Atherosclerotic Cardiovascular Disease		Risk of Cardiovascular Mortality	
	r	*p*	r	*p*
Age	0.596	0.000 *	0.508	0.000 *
Body mass index	0.111	0.361	0.094	0.437
Systolic blood pressure	0.280	0.019 *	0.421	0.000 *
Diastolic blood pressure	0.115	0.342	0.185	0.126
Smoking history	0.431	0.000 *	0.450	0.000 *
Total cholesterol	0.101	0.405	0.193	0.110
High-density lipoprotein	−0.337	0.004 *	−0.202	0.093
Serum creatinine	0.237	0.048 *	0.365	0.002 *
CKD stage	0.160	0.186	0.308	0.009 *
eGFR	−0.143	0.238	−0.284	0.017 *
Urine ACR	0.340	0.004 *	0.457	0.000 *
Plasma ACE	0.391	0.001 *	0.318	0.007 *

* Significant correlation at *p* < 0.05. ACE = angiotensin-converting enzyme; ACR = albumin/creatinine ratio; CKD = chronic kidney disease; eGFR = estimated glomerular filtration rate.

**Table 4:** Results of the correlational analysis between plasma ACE levels with the risk of ASCVD and cardiovascular mortality in each group of CKD.

CKD Groups	Plasma ACE Levels and ASCVD Risk		Plasma ACE Levels and Cardiovascular Mortality Risk	
	r	*p*	r	*p*
**Mild-moderate CKD**	0.319	0.048 *	0.16	0.332
**Severe CKD**	0.426	0.017 *	0.362	0.045 *

* Significant correlation at *p* < 0.05. ASCVD = atherosclerotic cardiovascular disease, mild–moderate CKD = eGFR > 30 mL/min/1.73 m^2^, severe CKD = eGFR < 30 mL/min/1.73 m^2^.

**Table 5:** Results of ACE I/D genotyping in CKD patients.

Genotype	*n*	Frequency (%)
II	43	61.4
ID	24	34.3
DD	3	4.3
Total	70	100
Recessive model	*n*	Frequency (%)
II	43	61.4
ID + DD	27	38.6
Total	70	100
Allele	*n*	Frequency (%)
I	110	78.6
D	30	21.4
Total	140	100

**Table 6:** Direct, indirect, and total effects of the path analysis.

Outcome	Direct Effect	Indirect Effect	Total Effect
Cardiovascular mortality risk			
ASCVD risk > Cardiovascular mortality risk	0.918 *		0.918 *
Plasma ACE level > Cardiovascular mortality risk	−0.06	0.376 *	0.316 *
ACE I/D polymorphism > Cardiovascular mortality risk	−0.019	−0.051	−0.071
ASCVD risk			
Plasma ACE level > ASCVD risk	0.41 *		0.41 *
ACE I/D polymorphism > ASCVD risk	−0.16	0.124 *	−0.036
Plasma ACE level			
ACE I/D polymorphism > Plasma ACE level	0.302 *		0.302 *

* *p* < 0.05.

## Data Availability

All relevant data are within the paper.
